# Physiological Response of *Corynebacterium glutamicum* to Increasingly Nutrient-Rich Growth Conditions

**DOI:** 10.3389/fmicb.2018.02058

**Published:** 2018-08-29

**Authors:** Michaela Graf, Julia Zieringer, Thorsten Haas, Alexander Nieß, Bastian Blombach, Ralf Takors

**Affiliations:** Institute of Biochemical Engineering, University of Stuttgart, Stuttgart, Germany

**Keywords:** *Corynebacterium glutamicum*, growth rate, substrate consumption, carbon and nitrogen consumption, amino acid uptake, stoichiometric network model *iMG481*, sensitivity analyses

## Abstract

To ensure economic competitiveness, bioprocesses should achieve maximum productivities enabled by high growth rates (μ) and equally high substrate consumption rates (q_S_) as a prerequisite of sufficient carbon-to-product conversion. Both traits were investigated and improved via bioprocess engineering approaches studying the industrial work horse *Corynebacterium glutamicum*. Standard minimal medium CGXII with glucose as sole carbon source was supplemented with complex brain-heart-infusion (BHI) or amino acid (AA) cocktails. Maximum μ of 0.67 h^-1^ was exclusively observed in 37 g BHI L^-1^ whereas only minor growth stimulation was found after AA supplementation (μ = 0.468 h^-1^). Increasing glucose consumption rates (q_Glc_) were solely observed in certain dosages of BHI (1–10 g L^-1^), while 37 g BHI L^-1^ and AA addition revealed q_Glc_ below the reference experiments. Moreover, BHI supplementation revealed Monod-type saturation kinetics of μ (*K*_BHI_ = 2.73 g BHI L^-1^) referring to the preference of non-AAs as key boosting nutrients. ATP-demands under reference, 1 g BHI L^-1^, and AA conditions were nearly constant but halved in BHI concentrations above 5 g L^-1^ reflecting the energetic advantage of consuming complex nutrient components in addition to “simple” building blocks such as AAs. Furthermore, *C. glutamicum* revealed maximum biomass per carbon yields of about 18 g_CDW_ C-mol^-1^ irrespective of the medium. In AA supplementation experiments, simultaneous uptake of 17 AAs was observed, maximum individual consumption rates determined, and L-asparagine and L-glutamine were distinguished as compounds with the highest consumption rates. Employment of the expanded stoichiometric model *iMG481* successfully reproduced experimental results and revealed the importance of *C. glutamicum*’s transaminase network to compensate needs of limiting AA supply. Model-based sensitivity studies attributed the highest impact on μ to AAs with high ATP and NADPH demands such as L-tryptophan or L-phenylalanine.

## Introduction and Motivation

*Corynebacterium glutamicum* is a Gram-positive, non-sporulating, facultative anaerobic prokaryote (Liebl, 2006; Nishimura et al., 2007), generally recognized as safe (GRAS), and best known for its production of L-glutamate (Kinoshita et al., 1957). Revelation of the *C. glutamicum* ATCC 13032 wild type (WT) genome by Ikeda and Nakagawa (2003) and Kalinowski et al. (2003) enabled application of metabolic engineering techniques and the construction of genome scale metabolic models (Kjeldsen and Nielsen, 2009; Shinfuku et al., 2009). Thereby, targeted modification of the strain’s genome, often predicted by said *in silico* models, enhanced *C. glutamicum*’s abilities to produce lactate, succinate, and pyruvate (Dominguez et al., 1993; Okino et al., 2005; Wendisch et al., 2006; Wieschalka et al., 2012), to form non-natural compounds such as diamins (Kind et al., 2010) or heterologous proteins (Scheele et al., 2013), and to emerge in the field of bio-based chemicals, materials, and fuels (Becker and Wittmann, 2012). Besides, the advantageous physiological properties, summarized by Vertès et al. (2012), further empower the microorganism to endure harsh production conditions as simulated and investigated in several scale-down studies mimicking production-like environments (Buchholz et al., 2014b; Käß et al., 2014a,b). Accordingly, the strain is well accepted as industrial platform and predominately employed for the production of the amino acids (AAs) L-glutamate, L-lysine, and L-valine (Hermann, 2003) serving demands of food, feed, cosmetic, and pharmaceutical markets (Takors et al., 2007). More than 2 million tons per year of L-glutamate (Becker and Wittmann, 2012) are likely to be produced by the strain in large scale bioreactors reaching volumes of 500 m^3^ and more.

Regarding industrial AA production, *C. glutamicum* competes with *Escherichia coli*. Whereas the first offers advantages in robustness also including minimized phage contamination vulnerability, *E. coli* naturally possesses transhydrogenases which allow quick equilibration of NADH/NAD^+^ and NADPH/NADP^+^ pools (Sauer et al., 2004). The latter is of particular importance to engineer top producers of NADPH demanding compounds such as L-lysine requiring 4 mol NADPH in the synthesis of 1 mol L-lysine (Marx et al., 1997). Besides, *C. glutamicum* reveals only second-best properties in terms of growth kinetics: maximum growth rates of about 0.58 h^-1^ have been measured for *C. glutamicum* strain ATCC 17965 in batch and continuous cultivations with minimal medium containing glucose (Cocaign-Bousquet et al., 1996; Gourdon et al., 2003) and in turbidostatic processes using *C. glutamicum* ATCC 13032 (Bäumchen et al., 2007). Grünberger et al. (2013) achieved even slightly faster growth rates of 0.60–0.64 h^-1^ in highly diluted microliter-scale conditions using CGXII minimal medium with glucose as sole carbon source. However, the same medium only shows approximately 0.4 h^-1^ in conventional bioreactor setups (Blombach et al., 2013; Grünberger et al., 2013). On the contrary, *E. coli* achieves maximum growth rates of up to 1.04 h^-1^ in glucose supplemented minimal medium and 1.73 h^-1^ in complex broth (Cox, 2004). In general, low growth rates inherently limit the productivity of the production process: first by achieving the desired biomass concentration, second by limiting product formation in growth coupled kinetics. By analogy, low biomass specific glucose uptake rates represent an intrinsic disadvantage of the producing cell as they may cause low biomass specific productivities, too.

Consequently, to increase the attraction of *C. glutamicum* for industrial application, improvement of fundamental kinetics would be highly appreciated. Strain and process engineering measures could be applied. The first, however, is beyond the scope of this contribution. Instead, properly applied medium supplements may improve productivities of existing production processes without necessary strain engineering. Accordingly, our contribution investigates process engineering means, in particular medium supplementation, for increasing growth and glucose consumption rates of *C. glutamicum* WT as a prerequisite of improving volumetric productivities. Industrial bioprocess development aims at reducing complex media such as yeast extract or corn steep liquor to reduce medium costs and variabilities. Of course, key performance criteria of the fermentation should still be achieved. Accordingly, the identification of so-called “goodies” of medium constituents is welcomed. However, the general physiological response of *C. glutamicum* to complex medium (compounds) was not studied yet. To date, supplementation investigations with the strain mostly aimed at media optimization (Coello et al., 2002; Jeon et al., 2013) or usage of alternative carbon and/or nitrogen sources (Lee et al., 2014; Wendisch et al., 2016; Lange et al., 2017). For *E. coli*, in contrast, profound knowledge exists based on physiological investigations in Luria–Bertani (LB) broth (Sezonov et al., 2007) and due to comparable transcriptional expression analyses of late-exponential *E. coli* cultures grown in minimal and LB medium (Tao et al., 1999). This approach was further expanded in a broad gene expression study analyzing the different growth phases of the enterobacterium grown in the same complex medium (Baev et al., 2006a,b,c).

Thus, we performed a two-staged analysis to study how supplementation of standard growth minimal medium CGXII impacts the physiology of *C. glutamicum* WT in bioreactor batch cultivations. In the first part, complex brain-heart-infusion (BHI) was added to CGXII to investigate and identify the maximum metabolic capacity of the cell regarding growth. As a step toward the identification of optimum synthetic growth medium, AA supplementation was studied in the second part which also allows the analysis of theoretical impacts via stoichiometric modeling. Until now, AA consumption was only investigated in transporter characterization studies (e.g., Burkovski et al., 1996; Trötschel et al., 2008; Shang et al., 2013) or under anaerobic conditions (Michel et al., 2015). In summary, the manuscript focuses on process engineering approaches to accelerate growth of *C. glutamicum*.

## Materials and Methods

### Experimental Setup

#### Preculture Cultivation

*Corynebacterium glutamicum* ATCC 13032 WT cells from working cell bank glycerol stocks were spread on 2 x tryptone-yeast extract (2 x TY) medium (Sambrook, 2001) agar plates and incubated for 48–60 h at 30°C. Colonies were used to inoculate 5 mL of 2 x TY medium in glass reaction tubes which were incubated for 8 h at 30°C and 120 rpm on a bench-top rotary shaker (Infors HT, Bottmingen, Switzerland). The content of one glass reaction tube was used to inoculate 50 mL modified CGXII minimal medium (Buchholz et al., 2014a) supplemented with 4% (w/v) glucose. The shaking flask precultures were incubated overnight at 30°C and 120 rpm.

#### Cultivation in Bioreactors

Bioreactor cultivations were carried out in two different bioreactor systems. Experiments with BHI-supplementation were conducted in biological triplicates in a parallel triplex glass bioreactor system (HWS Labortechnik, Mainz, Germany) with a working volume of 250 mL. CGXII minimal medium (Buchholz et al., 2014a) was supplemented with 2% (w/v) glucose (reference) and optionally 1, 5, 10, or 37 g BHI (1, 5, 10, 37 BHI) medium per liter. Culture broth from preculture shaking flasks was harvested, centrifuged at 4°C for 10 min at 4000 × *g* (5430 R, Eppendorf, Hamburg, Germany) and resuspendend in sterile 0.9% (w/v) NaCl-solution. The differently supplemented CGXII media were inoculated with the washed preculture installing initial optical densities (OD_600_) of 1 in 200 mL reaction volume. The parallel reactor system was equipped with dissolved oxygen (pO_2_)- and pH-probes (Mettler Toledo GmbH, Albstadt, Germany). The temperature was kept constant at 30°C, pO_2_ was kept >30% by increasing the impeller speed by 50 rpm (initial speed: 200 rpm) and the aeration rate by 20 mL min^-1^ (initial rate: 20 mL min^-1^) in a cascadic fashion. pH was kept constant by adding 25% (v/v) NH_4_OH. Antifoam agent (Struktol^®^J 647, Schill + Seilacher, Hamburg, Germany) was added manually when needed.

For AA supplementation experiments, biological triplicates were conducted in a 3 L steel-tank bioreactor (Bioengineering, Wald, Switzerland) ensuring high cell densities and enabling off-gas analysis. CGXII minimal cultivation media supplemented with 4% (w/v) glucose and optionally 5 mmol of the following AAs per liter were used: L-alanine (Ala), L-asparagine (Asn), L-aspartate (Asp), L-arginine (Arg), L-glutamine (Gln), L-glutamate (Glu), glycine (Gly), L-histidine (His), L-isoleucine (Ile), L-leucine (Leu), L-lysine (Lys), L-methionine (Met), L-phenylalanine (Phe), L-proline (Pro), L-serine (Ser), L-threonine (Thr), L-tryptophan (Trp), and L-valine (Val); 100 mL washed shaking flask preculture was used to inoculate supplemented CGXII media to give a starting OD_600_ of 3 and a total of 1.5 L reaction volume. The bioreactor was equipped with a six-blade Rushton impeller, a pO_2_-, and pH-probe and operated at a total pressure of 1.5 bar at 30°C. pO_2_ was kept > 30% by increasing the impeller speed by 50 rpm (initial speed: 250 rpm) and the aeration rate by 0.3 L min^-1^ (initial rate: 0.3 L min^-1^) in a cascadic fashion. pH was kept constant by adding 25% (v/v) NH_4_OH. Exhaust gas analysis of molar O_2_- and CO_2_-fractions was performed with non-dispersive (photometric) infrared gas analyzers (BCPO2 and BCPCO2, BlueSens, Herten, Germany). Antifoam agent was added manually when needed.

#### Analytical Methods

During the BHI-supplementation experiments, biosuspension samples were withdrawn hourly to measure the biomass concentration and to obtain cell-free samples. Determination of biomass was performed in technical replicates by following the optical density (OD_600_) of the cell suspension with a spectrophotometer (DR 3900, Dr. Lange, Berlin, Germany) at 600 nm. During the AA supplementation experiments, biomass was additionally measured gravimetrically: 1 mL biosuspension was centrifuged at 20,173 × *g* and 4°C for 2 min (5430 R, Eppendorf, Hamburg, Germany), washed twice with deionized water, and dried at 105°C for at least 24 h in a convection oven (Heraeus, Hanau, Germany). Correlating the OD_600_ and cell-dry-weight (CDW) measurements resulted in a correlation factor of 0.27 g biomass L^-1^. Cell-free samples were obtained by applying syringe filters with 0.2 μm pore size (Rotilabo^®^, Carl Roth, Karlsruhe, Germany) and storing the filtrates at -20°C until glucose-, total organic carbon- (TOC), and AA-quantification was performed. Glucose concentration was determined with an enzymatic assay following the instructions of the manufacturer (R-biopharma, Darmstadt, Germany). Following the protocols given by Buchholz et al. (2014a), AA concentrations were determined by means of high-pressure liquid chromatography, and total inorganic carbon (TIC)-, and TOC-amounts in biosuspension samples, cell-free filtrates, and biomass samples were measured with total carbon (TC) analysis. Therefore, a TC analyzer (Multi N/C 2100s, Analytik Jena, Jena, Germany) was employed to analyze samples regarding (i) the TIC content by acidification with 10% *ortho*-phosphoric acid to free carbonate-derived CO_2_ and (ii) the TC content by total combustion of organic and inorganic carbon species to CO_2_ at 750°C in a furnace system. TOC amounts were obtained with the differential detection mode of the device (TOC = TC – TIC). In the AA supplementation experiments, harvesting frequency was doubled after 4 h, and additional sampling enabled measuring of TOC and TIC values to check CO_2_ exhaust-gas analysis as proposed by Buchholz et al. (2014a).

### Determination of Kinetic Parameters

#### Exponential Growth Rate

Exponential growth rates (μ) were determined using linear regression of logarithmic biomass concentration curves over the process time as described by Grünberger et al. (2013) for batch cultivations.

#### Consumption Rates and Yields

Biomass-specific glucose, AA, and TOC consumption rates (q_Glc_, q_AA_, q_C_) were determined by dividing the exponential growth rate (μ) by the biomass substrate yield (Y_XS_) previously calculated by linear regression of substrate (S, representing glucose or carbon) concentrations curves over the biomass (X) concentration.

$$qs=\frac{\mu }{Y_{XS}}$$

#### Respiratory Rates in Amino Acid Supplementation Experiments

Biomass-specific respiratory rates (q_O2_, q_CO2_) from AA supplementation experiments were determined by dividing the volumetric oxygen consumption or carbon dioxide emission rate by the biomass concentration. q_CO2_ deduced from the off-gas analysis was corrected by accounting for dissolved carbon species in the liquid volume (TIC) in the early growth phase (Buchholz et al., 2014a).

#### Estimation of Respiratory Rates and ATP-Biomass Yield in BHI Supplementation Experiments

To estimate the produced CO_2_ during the BHI-supplementation experiments, molar (*n*) carbon (C) balancing was performed assuming that the consumed molar amount of TOC (n_C(TOC)_) in the time frame of the exponential growth phase was either converted to biomass or to CO_2_ neglecting TIC species accumulating in the fermentation broth in the early growth phase

$$\Delta n_{C\left({CO}_{2}\right)}=\Delta n_{C\left(TOC\right)}-\Delta n_{C\left(biomass\right)}$$

Determination of TOC in biomass and cell-free samples by TC-analysis allowed calculation of the theoretically produced amount of CO_2_. Thereof, the CO_2_-biomass yield was obtained and converted to q_CO2,est_ by multiplication with μ. The corresponding oxygen consumption rate q_O2,est_ was deduced from q_CO2,est_ by assuming a respiratory quotient (RQ) of 1, which was supported by the experimental rates of the reference and AA-supplementation experiments (c.f. **Tables 1**, **2**) in the steel-tank bioreactor

$$q_{O_{2},est}=RQ\cdot q_{{CO}_{2},est}$$

**Table 1:** Summary of kinetic parameters determined and estimated (index est) for *C. glutamicum* ATCC 13032 grown in modified CGXII minimal medium supplemented with 2% (w/v) glucose (Glc, reference) and additionally 1, 5, 10, or 37 g BHI L^-1^ (abbr. to 1, 5, 10, 37 BHI).

Supp.	μ,	q_Glc_,	q_C_,	Y_XC_	q_CO2,est_; $q_{O2est}^{*},$	q_ATP_,	Y_ATPX_,
	h^-1^	C-mol g^-1^ h^-1^	C-mol g^-1^ h^-1^	g C-mol^-1^	mmol g^-1^ h^-1^	mmol g^-1^ h^-1^	mmol g^-1^
Glc	0.32 ± 0.02	0.0214 ± 0.0015	0.0216 ± 0.0016	14.84 ± 0.33	6.94 ± 0.28	8.33 ± 0.34	26.03 ± 2.08
+1 BHI	0.44 ± 0.01	0.0277 ± 0.0016	0.0255 ± 0.0021	17.29 ± 1.34	8.79 ± 1.09	10.55 ± 1.31	23.90 ± 3.04
+5 BHI	0.54 ± 0.02	0.0272 ± 0.0014	0.0281 ± 0.0027	19.18 ± 1.64	5.48 ± 1.13	6.58 ± 1.35	12.22 ± 2.56
+10 BHI	0.59 ± 0.01	0.0264 ± 0.0007	0.0325 ± 0.0015	18.16 ± 0.73	6.32 ± 1.63	7.59 ± 1.95	12.87 ± 3.33
+37 BHI	0.67 ± 0.03	0.0191 ± 0.0019	0.0368 ± 0.0003	17.61 ± 1.61	8.13 ± 0.46	9.76 ± 0.56	14.67 ± 1.01


*Bioreactor batch cultivations were performed at 30°C and pH 7.4 in biological triplicates. Values represent the statistical mean ± standard deviation.*

*^∗^Estimated rates for oxygen consumption (q_O2,*est*_) and carbon dioxide emission (q_CO2,*est*_) are identical due to assumed respiratory quotient (RQ) of 1.*

**Table 2:** Summary of kinetic parameters determined for *C. glutamicum* grown in modified CGXII minimal medium supplemented with 4% (w/v) glucose (Glc) or additionally 5 mmol of 18 amino acids (AAs) per L (abbr. to 5 AA).

Supp.	μ,	q_Glc_,	q_C_,	Y_XC_	q_O2_,	q_CO2_,
	h^-1^	C-mol g^-1^ h^-1^	C-mol g^-1^ h^-1^	g C-mol^-1^	mmol g^-1^ h^-1^	mmol g^-1^ h^-1^
Glc	0.337 ± 0.033	0.0213 ± 0.0020	0.0213 ± 0.0020	15.82 ± 0.03	6.06 ± 0.48	6.17 ± 0.65
+5 AA	0.468 ± 0.003	0.0205 ± 0.0006	0.0257 ± 0.0006	18.00 ± 0.52	9.49 ± 0.85	8.92 ± 0.09


*Individual AA consumption rates are given in **Table 3**. Bioreactor batch cultivations were performed at 30°C, 1.5 bar, and pH 7.4 in biological triplicates. Values represent the statistical mean ± standard deviation.*

ATP production and consumption were assumed to be equilibrated. The first was deduced from q_O2,est_ assuming a P/O-ratio of 1.2 as a conservative estimate. Accordingly, the biomass-specific ATP production rate (q_ATP_) could be calculated as

$$q_{ATP}=P/O\cdot q_{O_{2},est}$$

In turn, division of q_ATP_ by μ resulted in the ATP-biomass yield Y_ATPX_. Standard deviations (SDs) for C-balancing are based on experimental results (biological triplicates) of carbon measurement of biomass and TOC, while SDs in all further calculation steps (cf. Eqs 3 and 4) were calculated by Gaussian error propagation.

#### Fitting of Monod-Type Parameters

The exponential growth rate μ of a microorganism can be calculated based on Monod-type kinetics (Monod, 1949)

$$\mu =\mu_{\max}\frac{c_{S}}{c_{S}+K_{S}}$$

where c_S_ is the substrate concentration of the only growth limiting substrate, K_S_ is the half-saturation constant (which equals the substrate concentration leading to the half maximum growth rate), and μ_max_ is the maximum growth rate observed for the used substrate. In this study, Monod-kinetics were applied to examine the effect of different BHI concentrations (1–37 g L^-1^) on the corresponding growth rate (μ_BHI_) by calculating the difference (Δμ) between growth rates obtained from experiments without BHI [μ_Ref_, 2% (w/v) glucose as only carbon source] and μ_BHI_

Δμ=μBHI−μRef

Inserting Eq. (6) into Eq. (5) depicted the influence of BHI-concentration (c_BHI_) on the resulting growth difference

$$\Delta \mu ={\Delta \mu }_{\max}\frac{c_{BHI}}{c_{BHI}+K_{BHI}}$$

To determine the unknown parameters Δμ_max_, that is the difference between the reference growth rate and the maximum growth rate observed under 37 g BHI L^-1^, and the saturation constant of BHI (*K*_BHI_), which is the BHI-concentration leading to half-maximum growth, the Curve Fitting Toolbox of Matlab (MATLAB Release 2014a, The MathWorks, Inc., Natick, MA, United States) was used. Thereby, non-linear least squares fitting to the experimental data based on the Monod-equation (7) were performed making use of the Levenberg–Marquadt algorithm and setting the confidence level to 90%.

### Simulations With Stoichiometric Network Model *iMG481*

#### Curation of Model *iEZ475* and Expansion to *iMG481*

The stoichiometric model curated and expanded in this work is based on model *iEZ475* of Zelle et al. (2015). Recently, another *C. glutamicum* reconstruction was crafted by Zhang et al. (2017), but was not considered in this investigation. After examination of AA biosynthesis and transporter reactions in *iEZ475*, the following alterations and expansions were performed according to published knowledge on the topics. *iMG481* is attached in the **Supplementary Material** as sbml- and Excel-file.

Regarding AA biosynthesis, the main route for Ala synthesis via alanine-transferase AlaT (Marienhagen and Eggeling, 2008) using pyruvate (Pyr) and Glu as precursors was added as reaction “alaT” yielding Ala and oxo-glutarate as products. Recently, Buerger et al. (2016) annotated gene *glsK* (*cg2728*) encoding the only known glutaminase in *C. glutamicum*. The corresponding hydrolyzation of Gln to Glu and NH_3_ was added as irreversible reaction “glsK.” Concerning the transport of AAs into the *in silico* cell, the direction of already implemented reactions “SER_t_NA” and “THR_t_NA,” formerly used for export of the AAs, was reversed to enable Ser and Thr import with Na^+^-symport (Palmieri et al., 1996) since the export of both AAs is independent of sodium ions (Palmieri et al., 1996). The Na^+^-flux, formerly in antiport with Gln, was reversed in reaction “GLN_t_NA” according to Siewe et al. (1995). Lys import can be facilitated by antiport with Ala, Ile, and Val (Bröer and Krämer, 1990) and reaction “LYS_t_LEU” which falsely uses Leu was corrected to “LYS_t_ILE” transporting Ile.

Incorporating *C. glutamicum*-specific information on AA transporters, several reactions were added: (i) Asn transport, hitherto only possible via diffusion, was expanded by a Na^+^-symporter (“ASN_t_NA”) making use of (supposedly) the same transporter as for Gln (Siewe et al., 1995); (ii) Asp uptake was expanded by ATP-dependency (“ASP_t_ATP”) mirroring the same transporter type as for Glu uptake (Krämer et al., 1990; Marin and Krämer, 2007); and (iii) Met transport facilitated by Na^+^-symport (Trötschel et al., 2008) was added (“MET_t_NA”) to the existing ATP-dependent system. No Arg-transporter was implemented in model *iEZ475* because Arg transport or a specific Arg transporter is not yet characterized or validated for *C. glutamicum*. However, Arg consumption was observed during the experiments (cf. section “Amino Acid Consumption and Amino Acid-Biomass Yield”), and therefore an ATP-dependent importer (“ARG_t_ATP”) was added based on experimental evidence for *E. coli* (Gong et al., 2003).

#### Flux Balance Analysis

Growth rates, AA consumption rates, and metabolic flux distributions of the *in silico* cell were calculated using the *optimizeCbModel* function of “The COBRA Toolbox” (Heirendt et al., 2017) with solver set “glpk” and Matlab software. Biomass production was set as objective function, while experimentally determined glucose consumption rates (q_Glc_), AA consumption rates (q_AA_), and respiratory rates (q_O2_, q_CO2_) were used as constraints. To simulate abundant AA supply, AA uptake was unconstrained by setting the upper flux bound to 1000.

#### Sensitivity Analysis

To study the impact of AA supplementation on the growth rate of the *in silico* cell, sensitivity analyses were performed. Thus, FBAs were conducted where the glucose uptake rate was constrained to 3.5 mmol g^-1^ h^-1^, representing the operation mode of exponential growth (cf. section “Amino Acid Consumption and Amino Acid-Biomass Yield”), and that of one single AA (q_AA_) to 3.5 nmol g^-1^ h^-1^ with the objective to maximize growth (μ_Sim_). FBA with only glucose uptake and without AA uptake yielded the reference growth rate μ_Ref_. The sensitivity of each AA was thus calculated as:

$$Sensitivity=\frac{\mu_{sim}-\mu_{ref}}{q_{AA}}$$

## Results

### BHI Supplementation Experiments

#### Kinetic Parameters

**FIGURE 1:**
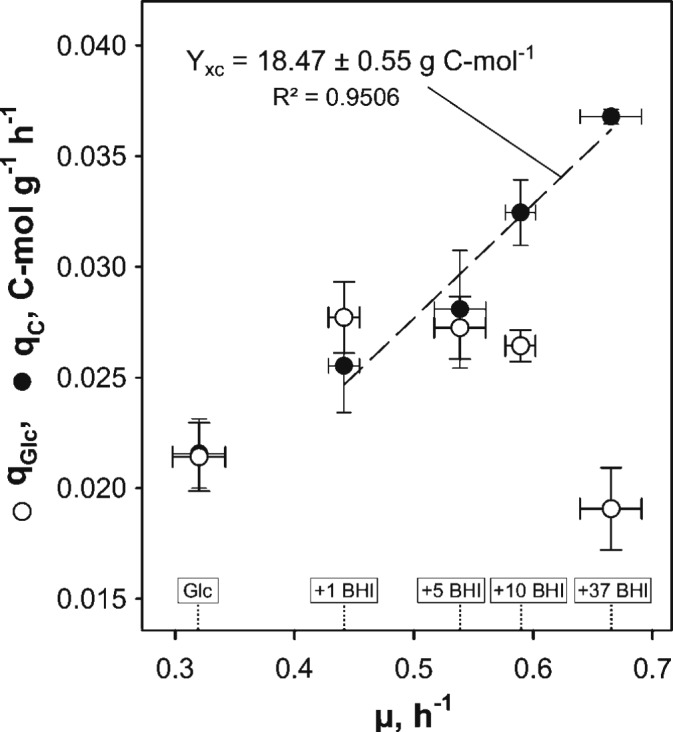
Illustration of biomass-specific glucose (q_Glc_) and total organic carbon (q_C_) consumption rates obtained in experiments with *C. glutamicum* WT grown in modified CGXII minimal medium with 2% (w/v) glucose (Glc) and brain-heart-infusion (BHI)-supplemented media (1, 5, 10, or 37 g BHI L^-1^) over the corresponding exponential growth rate (μ). A proportional increase of q_C_ in BHI-supplemented conditions with μ indicated a constant biomass-organic carbon yield (Y_XC_, cf. **Table 1**). Values are deduced from three biological replicates ± standard deviation.

The impact of BHI medium on growth behavior and kinetic parameters of *C. glutamicum* ATCC 13032 was studied in triplicate bioreactor cultivations. Therefore, 1, 5, 10, or 37 g BHI L^-1^ (1, 5, 10, 37 BHI) was added to modified CGXII minimal medium [2% (w/v) glucose] and the determined kinetic properties were compared to reference cultivations with glucose as sole carbon source. Calculated exponential growth rates μ obtained from each condition showed an increase of μ with increasing BHI amounts (μ = 0.44–0.67 h^-1^; **Table 1**). Under reference conditions, μ = 0.32 ± 0.02 h^-1^ was observed and a maximum rate of 0.67 ± 0.03 h^-1^ in 37 BHI. Pairwise *t*-tests between conditions with the most similar μ confirmed significant differences in growth mirroring the different BHI supplements. Besides, BHI stimulated the glucose consumption under 1–10 BHI conditions, revealing q_Glc_ of 0.0277 ± 0.0016 C-mol g^-1^ h^-1^ in 1 BHI, which was 29% higher than the reference. However, a decline of q_Glc_ with increasing growth rate promoted by increasing BHI amounts was observed (**Figure 1**, open circles), finally reaching a value of 0.0191 ± 0.0019 C-mol g^-1^ h^-1^ in 37 BHI that was 11% smaller than in reference conditions. In contrast to glucose consumption, the TC consumption rate q_C_ increased proportionally with μ as illustrated in **Figure 1** (filled black circles). Thereby, a constant biomass-carbon yield (Y_XC_) of 18.47 ± 0.55 g C-mol^-1^ could be calculated by linear regression of biomass production versus carbon consumption.

#### Monod-Like Kinetics

**FIGURE 2:**
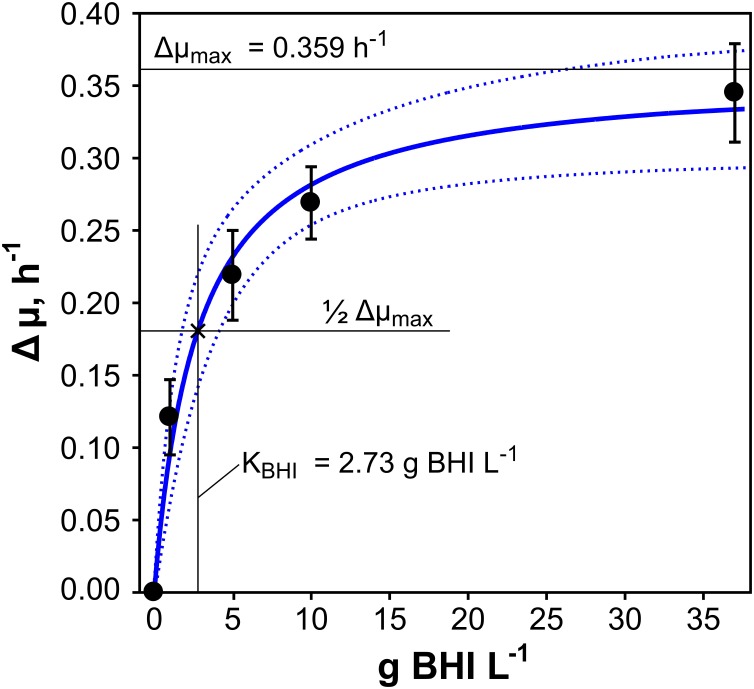
Exponential growth rate differences (Δμ) were calculated for *C. glutamicum* WT grown in modified CGXII minimal medium supplemented with 2% (w/v) glucose and with additional 1, 5, 10, or 37 g brain-heart-infusion (BHI) L^-1^ medium (three biological replicates ± standard deviation). Illustration of Δμ over the corresponding amount of BHI revealed a Monod-like saturation curve. The half-velocity constant for BHI (*K*_BHI_) and the maximum growth rate difference (Δμ_max_) given in the figure were deduced from non-linear least-squares fitting (blue solid line) with a confidence level of 90% (blue dotted lines).

The supplementation of CGXII minimal medium with different amounts of BHI induced a diversification of the growth rate of *C. glutamicum* which motivated us to investigate the influence of BHI concentration on μ. Therefore, differences in growth (Δμ) of BHI-supplemented and non-supplemented conditions (reference) were calculated (Eq. 6) and plotted against related BHI concentrations (1–37 g BHI L^-1^), illustrated in **Figure 2** (filled black circles). A Monod-like saturation curve was observed with a steep increase of Δμ for low BHI additions (1–5 g BHI L^-1^) leveling out at BHI-concentrations above 10 g L^-1^ and approximating the maximal growth rate difference (Δμ_max_). Therefore, non-linear least squares fitting of the experimental data was performed, using the Monod-type equation (7) with 90% confidence level of the fit. Δμ_max_ and the saturation constant of BHI (*K*_BHI_), that is the BHI concentration leading to half-Δμ_max_, were thus estimated with 0.359 h^-1^ and 2.73 g BHI L^-1^, respectively. Plugging both values into Eq. 7 results in the estimation curve (blue solid line) pictured in **Figure 2** with the dotted curves above and below as a measure for the confidence level.

#### Estimated ATP-Biomass Yield

**FIGURE 3:**
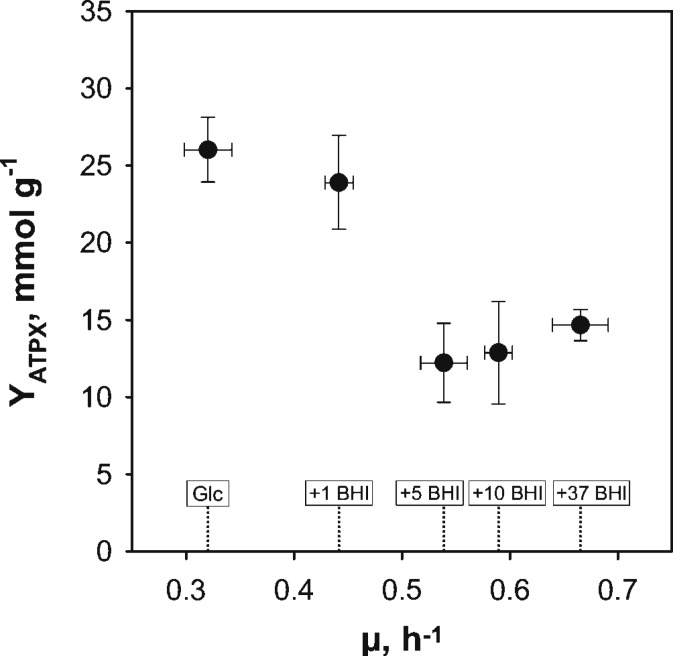
Illustration of estimated ATP-biomass yields (Y_ATPX_) over exponential growth rates (μ) from BHI-supplementation experiments with *C. glutamicum* WT grown in modified CGXII minimal medium with 2% (w/v) glucose (Glc) and brain-heart-infusion (BHI)-supplemented media (1, 5, 10, or 37 g BHI L^-1^). Y_ATPX_-estimation was based on carbon balancing to calculate the theoretical respiratory rates and assuming a P/O-ratio of 1.2 (cf. Eqs 2–4 and **Table 1**). Values are deduced from three biological replicates and error propagation was used to determine corresponding standard deviations pictured as error bars in the figure.

The ATP consumption of *C. glutamicum* cultivated in supplemented CGXII media was estimated to evaluate the energy demand of the cell under minimal and increasingly rich conditions. Since no intracellular ATP pools were measured, the theoretical amount of ATP produced and consumed by the strain was estimated as given in Eqs 2–4. Therefore, carbon balancing was performed to estimate the produced molar amount of CO_2_ which was converted to the (estimated) carbon emission rate q_CO2,est_ (**Table 1**). Assuming a RQ of 1 led to the corresponding oxygen consumption rate q_O2,est_, which was then used to calculated the ATP consumption rate by assuming a P/O ratio of 1.2. The resulting ATP-biomass yield Y_ATPX_ is plotted against growth rates in **Figure 3**. Under reference conditions (glucose as sole carbon source), the highest ATP-yield of 26.03 ± 2.08 mmol g^-1^ was observed, whereas the yield with 1 g BHI L^-1^ was in the same range (23.9 ± 3.04 mmol g^-1^). However, elevation of BHI-amounts above 1 g L^-1^ lead to a new ATP-level of around 14 mmol g^-1^ considering the SDs of the respective conditions (5, 10, and 37 BHI). This equals a drop of the ATP-biomass yield by ca. 46% in comparison to reference conditions.

### Amino Acid Supplementation Experiments

#### Kinetic Parameters

To unravel whether the stimulative effect of BHI on growth rate and TC consumption of *C. glutamicum* WT was due to AAs, which are components of BHI in free or peptide-bound form, AA supplementation fermentations were performed. Thus, 5 mM of all essential AAs (except for L-cysteine and L-tyrosine) were added to modified CGXII minimal medium [4% (w/v) glucose] to comparably study the kinetic properties of the strain cultivated in the “simulated” complex medium. Impacts of elevated biomass concentrations were studied in the pressurized bioreactor to prevent oxygen limitation. To ensure comparability, reference cultivations with glucose as only carbon source were repeated in this bioreactor. Deduced kinetic parameters (**Table 2**) showed good conformity with corresponding values obtained in the previous system (**Table 1**). Accordingly, all results can be discussed irrespective of the reactor system. Addition of AAs to CGXII minimal medium increased μ by about 40% from 0.337 ± 0.033 (reference) to 0.468 ± 0.003 h^-1^ (**Table 2**) which was within the range of 1–5 BHI supplementation (μ = 0.44–0.54 h^-1^; **Table 1**). Interestingly, the maximum observed growth rate (0.67 ± 0.03 h^-1^) in 37 BHI was not reached by AA supplementation. As previously observed in 37 BHI, q_Glc_ determined under AA-supplemented conditions was within the range of the reference considering the deviance (AA: 0.0205 ± 0.0006 C-mol g^-1^ h^-1^; reference: 0.0213 ± 0.0020 C-mol g^-1^ h^-1^). In contrast, TC consumption was about 21% higher (0.0257 ± 0.0006 C-mol g^-1^ h^-1^) making it comparable to the values observed in 1 BHI (0.0255 ± 0.0021 C-mol g^-1^ h^-1^). Determination of the biomass-carbon yield Y_XC_ in AA supplementation revealed a value of 18.00 ± 0.52 g C-mol^-1^ which fits to the linear regression of Y_XC_ from the BHI-experiments (18.47 ± 0.55 g C-mol^-1^). Calculation of respiratory rates deduced from exhaust gas analysis displayed an increase of about 45% compared to the reference (q_O2_ = 6.06 ± 0.48 mmol g^-1^ h^-1^; q_CO2_ = 6.17 ± 0.65 mmol g^-1^ h^-1^) which equals the increase of growth (about 40%).

#### Amino Acid Consumption and Amino Acid-Biomass Yield

**FIGURE 4:**
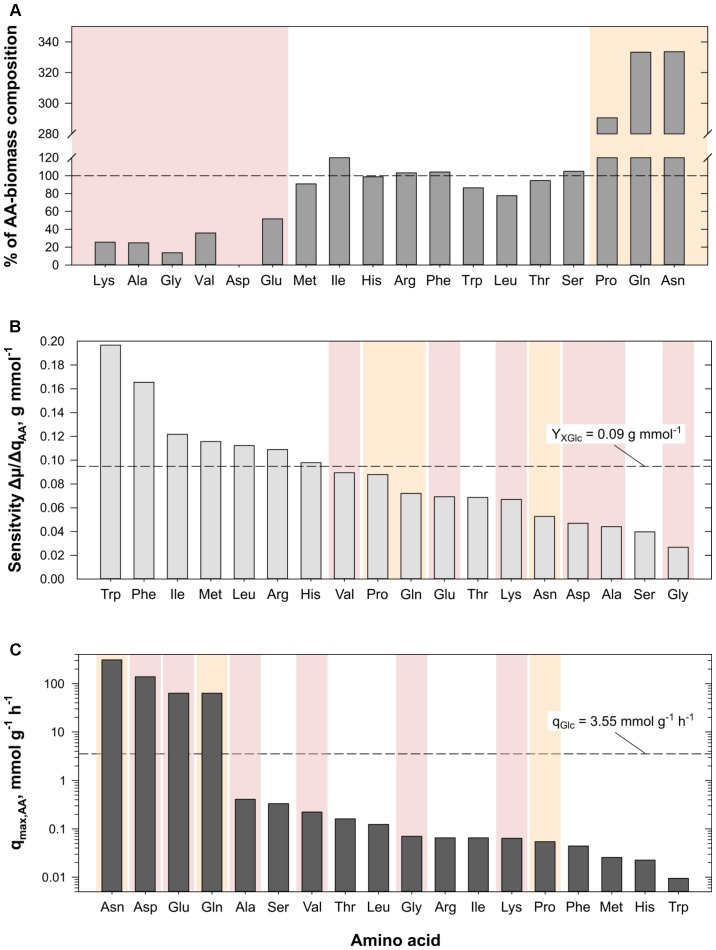
**(A)** Illustration of percentage amino acid (AA)-biomass yields (mmol g^-1^) calculated from AA supplementation experiments in relation to the mean AA-composition of biomass from different *C. glutamicum* strains (cf. **Table 4**). AA-amounts consumed during the experiments which were higher (above 100%) or lower (below 100%) than the published values are highlighted in yellow and red, respectively. **(B)** Results of the sensitivity analyses indicating the impact of AA-uptake on the growth rate of the *in silico* cell (cf. Eq. 8). The experimental biomass-glucose yield (Y_XGlc_) observed for the strain grown in CGXII [4% (w/v) glucose, **Table 2**] is given as reference. Color-coding is adapted from panel (A). **(C)** Results of unbound AA-uptake (q_max,AA_) by the *in silico* cell. The experimental glucose consumption rate (q_Glc_) observed for growth in CGXII [4% (w/v) glucose, **Table 2**] is given as reference. Color-coding is adapted from panel (A).

Regarding the consumption of the 18 added AAs in the AA supplementation experiments, all AAs except for Asp were consumed (cf. **Supplementary Figure S1**) by *C. glutamicum* WT. Simultaneous decrease of AA concentrations in cell-free filtrates was observed but for Asn which was consumed only after Gln was nearly depleted (3–4 h, **Supplementary Figure S1**). Related AA consumption rates were calculated for each AA and are summarized in **Table 3**. The highest rate of 0.694 ± 0.005 mmol g^-1^ h^-1^ was obtained for Gln, whereas Trp was consumed with the smallest rate (0.022 ± 0.001 mmol g^-1^ h^-1^). Next, AA-biomass yields (Y_AA_) were determined and compared to three biomass AA-compositions of different *C. glutamicum* species (**Table 4**): *Micrococcus glutamicus* No. 541 (*C. glutamicum* ATCC 13058, Kimura, 1963), *C. glutamicum* ATCC 17965 (Cocaign-Bousquet et al., 1996), and *C. glutamicum* MH20-22B (lysine producer based on *C. glutamicum* ATCC 13032, Marx et al., 1996). Mean biomass compositions were derived to check whether experimental Y_AA_ mirror the apparent needs for biomass build-up (last column in **Table 4**). Thereby, three groups could be identified: (i) AAs fitting well with the reference data (Arg, His, Ile, Leu, Met, Phe, Ser, Thr, Trp), (ii) AAs taken up in higher (Gln, Asn, Pro), or (iii) in lower amounts (Ala, Glu, Gly, Lys, Val, Asp) than expected from composition. **Figure 4A** illustrates the observation by color-coding AAs of group (i) in white, AAs of group (ii) in yellow, and AAs of group (iii) in red. Accordingly, consumed amounts of AAs of group (ii) exceed the biomass need approximately by factor three. AAs from group (iii) poorly satisfied the composition-demand between 0 (Asp) and 52% (Glu).

**Table 3:** Amino acid (AA) consumption rates (q_AA_) of *C. glutamicum* cultivated in CGXII minimal medium supplemented with 4% (w/v) glucose and 5 mM of all AAs except L-cysteine and L-tyrosine.

AA	q_AA_, mmol g^-1^ h^-1^
Gln	0.694 ± 0.005
Asn	0.302 ± 0.003
Pro	0.218 ± 0.010
Ser	0.134 ± 0.003
Thr	0.118 ± 0.001
Glu	0.117 ± 0.001
Leu	0.112 ± 0.002
Ile	0.108 ± 0.002
Arg	0.093 ± 0.003
Ala	0.083 ± 0.002
Phe	0.066 ± 0.002
Val	0.051 ± 0.008
Met	0.046 ± 0.002
His	0.031 ± 0.002
Gly	0.025 ± 0.006
Lys	0.025 ± 0.001
Trp	0.022 ± 0.001
Asp	n. c. d.


*Table is ordered according to the magnitude of q_AA_. Values represent the statistical mean ± standard deviation of three individual bioprocesses. n.c.d., no consumption detected.*

**Table 4:** Comparison of published amino acid (AA)-compositions of different *C. glutamicum* strains determined by (A) Kimura, 1963, (B) Cocaign-Bousquet et al., 1996, (C) Marx et al., 1996, and experimentally determined AA-yields (Y_AA_).

AA	AA compositions of *C. glutamicum* μmol g_CDW_^-1^					
				
				Mean or range of A, B, C	Experimental yield	Y_AA_/M,
	A	B	C	M ± SD, μmol g_CDW_^-1^	Y_AA_, μmol g_CDW_^-1^	%
Ala	802	725	606	711 ± 99	177 ± 5	25
Arg	196	190	189	192 ± 4	198 ± 8	103
Asn	n.v.	187	200	187–200 (194)	646 ± 4	334
Asp	434	187	200	187–200 (194)	n.c.d.	n.c.d.
Glu	569	486	403	486 ± 83	1482 ± 3	52
Gln	n.v.	486	403	403–486 (445)	251 ± 2	333
Gly	411	344	361	372 ± 35	51 ± 13	14
His	135	63	71	63–71 (67)	66 ± 5	99
Ile	535	175	202	175–202 (189)	231 ± 5	123
Leu	354	262	0	262–354 (308)	239 ± 6	78
Lys	260	171	202	211 ± 45	54 ± 3	26
Met	72	5	146	72–146 (109)	99 ± 4	91
Phe	148	123	133	135 ± 13	140 ± 5	104
Pro	163	148	170	160 ± 11	466 ± 23	290
Ser	326	265	225	272 ± 51	285 ± 7	105
Thr	252	270	275	266 ± 12	251 ± 1	95
Trp	n.v.	1	54	54	47 ± 3	86
Val	353	262	284	300 ± 47	107 ± 18	36


*For better comparison, the percentage of Y_*AA*_ in relation to the mean value (M) or value range (M in brackets) of A, B, and C ± standard deviation was calculated. Y_*AA*_-values represent the statistical mean ± standard deviation of three biological triplicates. n.v., no value available; n.c.d., no consumption detected.*

#### *In silico* AA-Uptake and -Consumption Studies With *iMG481*

##### Validation of stoichiometric network model *iMG481*

Besides studying the *in vivo* AA consumption of *C. glutamicum* WT during bioprocesses, *in silico* AA uptake and its influence on growth was further analyzed with the expanded stoichiometric network model *iMG481*. To check the suitability of the model, FBA was performed for reference conditions using q_Glc_ = 3.55 mmol g^-1^ h^-1^ as input, leaving q_O2_ and q_CO2_ non-constrained. μ_Sim_ = 0.337 h^-1^ was the output which agrees well with the experimental value (**Table 2**) and with μ_Sim_ obtained from the predecessor model *iEZ475* (Zelle et al., 2015). Because *iMG481* was derived from *iEZ475* by the implementation of additional AA importers, measured AA consumption rates, in particular q_Arg_, q_Ser_, and q_Thr_, could be used as additional inputs yielding μ_Sim_ = 0.481 h^-1^, which was slightly higher than the experimental μ (0.468 ± 0.003 h^-1^). Constraining respiration of the *in silico* cell to the experimental values reduced the predicted growth rate by ca. 20% in all tested cases. Therefore, following investigations were performed with unconstrained oxygen consumption or carbon dioxide emission.

Comparing predicted intracellular fluxes of *iEZ475* with *iMG481* under reference conditions revealed that the new reaction “alaT” (using Glu as amino donor for Ala-synthesis) was used by *iMG481* instead of the alternative route “NCgl0388” using Val as amino donor. This result was in accordance with Marienhagen and Eggeling (2008) describing the former reaction as the main synthesis route for Ala in *C. glutamicum*. Furthermore, under AA supplementation conditions, the new Gln hydrolysis reaction “glsK” was used by the *in silico* cell instead of reactions “glmS” and “pyrG_2gln” which, likewise to “glsK,” produce Glu from exceeding amounts of Gln.

Apart from the two new synthesis pathways, *iMG481* has a broader transport variability than *iEZ475* by incorporation of additional transporters for Ala, Asp, and Met. Accordingly, different uptake options for Ala, Asn, Asp, Glu, Ile, Leu, Met, Phe, and Val were available which were tested with regard to the preferred uptake system of the *in silico* cell. When AA uptake was unconstrained and at least one of the said AA was assumed to be active, the highest μ_Sim_ was always achieved by the least-energy demanding uptake system. The ranking found that diffusion was preferred to ion exchange assigning ATP-requiring uptake the least attraction. For further investigations, all transporters which were supposed to be active under non-limited substrate conditions were considered (**Supplementary Table S1**).

##### Sensitivity analysis: impact of AA uptake on μ

The impact of AA supplementation on the simulated growth rate μ_Sim_ was studied by means of sensitivity analysis. Substrate consumption rates under exponential growth conditions were taken as the reference. The impact on growth increase was investigated by adding infinitesimal amounts of singular AAs with 3.5 nmol g^-1^ h^-1^ which represented 1/1000 of the glucose uptake rate constraint. Thereby, the AA-related sensitivity on growth induced by each of the 18 AAs was obtained employing Eq. (8). **Figure 4B** ranks the growth sensitivities of these AAs giving the experimental biomass-glucose yield (Y_XGlc_, **Table 2**) as reference (dashed line). As a result, Trp, Phe, Ile, Met, Leu, Arg, and His showed sensitivities above the reference and therefore had the highest impact on μ_Sim_. Interestingly, this group of AAs was already identified in the experimental approach as AAs being consumed according to the AA-biomass composition (cf. **Table 4**), as highlighted by the color scheme adopted from **Figure 4A**. *Vice versa*, AAs exerting non-growth coupled impact on μ_Sim_ (e.g., Pro, Glu, Gln, etc.) were either consumed in lower or higher than expected amounts taking cellular AA-biomass composition as threshold.

##### Unbound AA-uptake

Complementary to the sensitivity analysis, the consequence of extreme AA uptake has also been studied. The simulations considered constant glucose uptake (3.5 mmol g^-1^ h^-1^) and eliminated any upper limit of AA uptake. The results pictured in **Figure 4C** show that Asn, Asp, Glu, and Gln surpassed the molar uptake of glucose (dashed line) by up to 85 times. Calculating the AA-biomass yield with the help of the simulated AA uptake and corresponding growth rate according to Eq. (1) (data not shown) further revealed that apart from the four mentioned AAs, Ala, Leu, Ser, Thr, and Val were taken up in higher amounts than expected from the model’s AA-biomass composition (cf. reaction “PROTEIN_a”). Gly, however, was the only AA with a taken-up amount below the composition value. Comparison of these AAs to the experimental AA-grouping and color scheme of **Figure 4A** identifies these AA (apart from Ser) as AAs exhibiting non-proportional uptake in the experiments.

## Discussion

The study was motivated by the fundamental question to elucidate the physiological response of *C. glutamicum* ATCC 13032 on increasingly nutrient-rich growth conditions. Therefore, a two-staged supplementation analysis was performed. In the first part, standard minimal medium CGXII with glucose as sugar source was supplemented with varying amounts of BHI to identify the cellular maximum of metabolic capacity. Since AAs are crucial components of BHI (in free or peptide-bound form), their influence on growth was analyzed in the second part of the study by adding an AA-cocktail comprising 18 AAs with individual concentrations of 5 mM. Using AA supplementation in synthetic medium enabled further *in silico* FBA studies. Both BHI and AA supplementation are discussed in the following.

### Impact of BHI and AAs on Growth and Carbon Consumption of *C. glutamicum*

Previous growth studies by Grünberger et al. (2013) showed that the strain achieved maximum growth rates of 0.42 ± 0.03 h^-1^ when cultivated in lab-scale bioreactors using CGXII. Thereof, Unthan et al. (2014) identified the supplement protocatechuic acid (PCA) in CGXII as growth-accelerating compound. Strictly speaking, it is not yet fully unraveled whether PCA serves as additional carbon source, affects iron transport, enhances growth by a combinatorial effect, or may even support metabolism by other means. Accordingly, CGXII minimal medium without PCA was used in our investigations. As expected, growth rates of 0.32 ± 0.02 h^-1^ were observed in non-supplemented conditions which could be increased to 0.45 ± 0.01 h^-1^ when PCA was used (data not shown). In the following, CGXII always implies PCA-free minimal medium with glucose as standard carbon source.

Adding varying amounts of BHI (1–37 g BHI L^-1^) to CGXII [2% (w/v) glucose] significantly increased μ from 0.32 ± 0.02 h^-1^ to a maximum of 0.67 ± 0.03 h^-1^ revealing Monod-type dependence of the growth rate increase (Δμ_max_) on BHI-concentration. Interesting enough, *K*_BHI_ of 2.73 g L^-1^ indicates the existence of a growth boosting component in BHI that is crucial for growth acceleration in low amounts but achieves saturating levels in BHI > 5 g L^-1^.

To elucidate whether the growth enhancement was due to AAs present in BHI, 18 AAs were added to CGXII [4% (w/v) glucose]. To ensure maximum AA uptake rates, abundant AA concentrations of 5 mM were installed which were sufficiently higher than published K_M_-values of the strain’s AA transporters (cf. **Supplementary Table S1**). Hence, an equally high growth rate of about 0.6 h^-1^ as induced by 37 g BHI L^-1^ was expected, if AAs were the only growth enhancing factors in BHI. Surprisingly, a remarkably smaller exponential μ_max_ of 0.468 ± 0.003 h^-1^ was observed which equaled the addition of 2 g BHI L^-1^ (**Figure 2**). However, quantification of free and peptide-bound AAs in BHI (data not shown) revealed that only about 8 mM AAs were available in 2 g BHI L^-1^ which was significantly lower than the total amount used in the AA experiments (90 mM). Since these findings indicated that AAs were not the only growth enhancing factors in BHI, we evaluated the findings by further energetic analysis.

Therefore, we qualified the substrates by estimating the ATP-biomass yields of the different nutrient scenarios. Assuming that total consumed carbon (supplied by glucose and BHI) was used to produce biomass, CO_2_, and no by-products, CO_2_ emission rates and O_2_ consumption rates were calculated (**Table 1**) and compared to experimentally determined rates (**Table 2**) of the glucose reference conditions. ATP formation was calculated assuming a growth medium-independent P/O-ratio of 1.2 which represents a realistic estimation based on own observations. Moreover, we presumed that ATP formation equaled consumption. Accordingly, the ATP-yield of about 26 mmol g^-1^ for reference conditions was observed (**Table 1**). The estimation fits well with findings of Cocaign-Bousquet et al. (1996) (29.1 mmol g^-1^), but differs from the 60 mmol g^-1^ determined by Pfelzer (2016). For comparison, *E. coli* shows demands of 71 mmol g^-1^ (Varma et al., 1993). However, estimations strictly depend on the choice of P/O-ratio. For example, choosing the P/O ratio of two yields 43 mmol g^-1^. Accordingly, estimated Y_ATPX_ values only show tendencies allowing comparison of the different media.

Almost identical Y_ATPX_ of 23.9 ± 3.04 and 24.3 ± 2.2 mmol g^-1^ were observed for the addition of 1 g BHI L^-1^ and AA supplementation, respectively. The observation somewhat mirrors the Monod-type growth increase after BHI addition (**Figure 2**) compared to the growth rise after AA supplementation. Both revealed similar growth benefits, apparently resembling similar energetic status of the cells. However, adding BHI ≥ 5 g L^-1^, steadily decreased Y_ATPX_ to about 14 mmol g^-1^ which corresponds to a 46% drop with respect to the reference. Several hypothesis can be formulated: (i) under reference, 1 g BHI L^-1^-, and AA-supplemented conditions, ATP demanding futile cycles existed which disappeared under BHI-rich conditions; (ii) P/O ratios are not constant but increase under BHI-rich conditions which would compensate the Y_ATPX_ drop; and (iii) BHI-rich media provide crucial precursors, thereby preventing own ATP-demanding cellular synthesis. Hypothesis (i) appears unlikely qualifying a 46% share of ATP production for futile cycling production as too high. Hypothesis (ii) requires for detailed (^13^C based) metabolic flux analysis which is beyond the scope of this study. Ruling out options (i) and (ii), hypothesis (iii) was taken as the guiding idea for subsequent analysis.

Accordingly, net ATP costs for AA biosynthesis starting from central metabolism precursors were estimated modifying the approach of Kaleta et al. (2013) who calculated the net ATP costs for AA synthesis routes in *E. coli* by considering ATP costs in the synthesis pathways but also accounted for the amount of produced ATP by precursor synthesis (cf. **Table 4** and **Supplementary Tables S2**, **S3**). In contrast to Kaleta et al. (2013), NADPH was not considered as energy (ATP) yielding precursor in respiration of *C. glutamicum* because the strain does not possess nicotinamide nucleotide-dependent transhydrogenases like *E. coli* (Kabus et al., 2007). As a result, ATP costs of about 4 mmol for 1 g biomass were calculated that represents less than 15% of the net ATP formation from glucose (about 26 mmol g^-1^, **Table 1**) considering the P/O ratio of 1.2. Accordingly, enriching the medium by AAs did create only minor energetic benefit as shown for the AA supplemented medium compared to the reference (difference of approximately 2 mmol g^-1^). Studies of Ingraham et al. (1983) analyzing ATP demands of *E. coli* grown in minimal and rich medium further support the observation: ATP demands of protein formation outnumber those of AA formation by far. Similar to our findings for *C. glutamicum*, *E. coli* revealed 40% reduced Y_ATPX_ values when cultivated in rich medium compared to minimal medium (Ingraham et al., 1983). Apparently, the reduction of 46% observed for *C. glutamicum* agrees well. Most likely, other ATP-demanding building blocks, such as fatty acids or nucleotides, are consumed in highly concentrated BHI environments which relieves the cellular need to create ATP thereby reducing the glucose uptake accordingly. Therefore, the growth phenotype resulting from different nutrient supplies can be explained as follows: *C. glutamicum* benefits from the abundant addition of AAs by omitting the related AA biosynthesis which yields a moderate μ increase. Growth advantages of AA supplementation may be rooted in the elimination of kinetics limits. For instance, external supply of AAs may fuel intracellular transaminases thereby preventing own “slow” biosynthesis. Alternatively, external supply may meet short-term needs for charging tRNAs as a prerequisite of fast translation for protein formation (Nieß et al., 2017). Adding high amounts of BHI further released the cell of ATP-costly fatty acid and nucleotide synthesis enhancing μ to its apparent limit of 0.67 h^-1^. Necessary ATP production was reduced to about 14 mmol ATP per g biomass resembling the remaining cellular needs for maintenance and biosynthesis. The strong growth boosting effect of 1 g BHI L^-1^ (**Figure 2**) anticipates that other growth stimulating components beside AAs (about 4 mM, cf. above) play a role, too. Likely candidates are trace elements or vitamins. The latter have been tested (supplementation of 0.2 mg thiamine-HCl L^-1^ in CGXII) and an elevated growth rate of approximately 0.4 h^-1^ was determined (data not shown).

Biomass specific glucose uptake rates q_Glc_ also showed particularities for different nutrient-rich media. Whereas 1 g BHI L^-1^ caused q_Glc_ increase, further BHI supplementation yielded decreasing rates (**Table 1**) reaching reference-like levels in 37 g BHI L^-1^ and in the AA supplementation experiments. Notably, TC consumption increased proportionally with μ showing a biomass-carbon yield Y_XC_ of 18.47 g C-mol^-1^. The observations agree with findings of Dominguez et al. (1997) who found Y_XC_ of 17.6 g C-mol^-1^ for the consumption of different glucose-fructose mixtures. Wendisch et al. (2000) observed 12 g C-mol^-1^ for the consumption of glucose-acetate mixtures and Frunzke et al. (2008) measured 13 g C-mol^-1^ for glucose, gluconate, and glucose–gluconate mixtures in shaking flask cultivations. Our study somewhat differs from the others by considering not only carbon uptake alone but also the parallel consumption of nitrogen containing AAs. Consequently, the maximum value of 18.47 g C-mol^-1^ may reflect the total cellular capacity limit of processing carbon and nitrogen (via AAs) through metabolism and translation, whereas Y_XC_ of 17.6 g C-mol^-1^ mirrors limits of sugar uptake and metabolism. Interestingly, both values are similar which hints to optimized carbon-to-biomass conversion irrespective of the medium composition and to equally adjusted capacities for AA processing, i.e., protein formation.

### Amino Acid Consumption of the *in vivo* and *in silico* Cell

The addition of an abundant supply of 18 AAs in minimal medium revealed three groups of AAs with distinctive consumption characteristics. AAs of the first group, consisting of Arg, His, Ile, Leu, Met, Phe, Ser, Thr, and Trp, were consumed according to AA-biomass composition. Biosynthesis pathways of most of these AAs, e.g., Met, Trp, or Phe, are energy-intensive, i.e., they have a positive net ATP-cost (**Supplementary Table S3**). Consequently, the cell safes energy when the AAs are consumed instead of being synthesized. The experimental observation agrees well with *in silico* analyses: growth rate-sensitivity studies revealed that the same AAs (apart from Thr and Ser) had the strongest influence on μ. Except for serving as AA building block like the other AAs, Ser may serve as carbon source in the absence of other available C-sources (Netzer et al., 2004). It is a precursor for synthesis of activated C_1_-compounds and for L-cysteine synthesis (Wada and Takagi, 2006). The latter was not supplemented in the experiments.

The second group consisted of Asn, Gln, and Pro showing consumption rates more than threefold higher than expected from the AA-biomass composition. In contrast, the third group of AAs (Ala, Asp, Glu, Gly, Lys, Val) was consumed remarkably below the expected threshold. The consumption rate of Gln was the highest of all AAs (0.694 mmol g^-1^ h^-1^) and approximated the maximum uptake rate of the Gln transporter (Siewe et al., 1995; **Supplementary Table S1**). Notably, Gln may serve carbon and nitrogen demands at the same time being decomposed by hydrolysis or deamination via glutaminase Glsk or comparable enzymes (Buerger et al., 2016). Glu and ammonia are the products which correlates increased Gln uptake with the reduced need of Glu and ammonia consumption. In case of Glu, the inadequate consumption of the AA might have been caused by the presence of glucose in the medium causing catabolite repression (Krämer et al., 1990; Kronemeyer et al., 1995). Likewise, the aforementioned compensation via Gln uptake may explain the observed low Glu consumption rates. Taking Gln instead of Glu also saves ATP which would have been needed when Gln had to be synthesized from Glu via Gln synthase. Irrespective whether Glu is produced from Gln, taken up, or synthesized from alpha ketoglutarate via glutamate dehydrogenase, Glu may serve as amino donor in transaminase reactions such as aspartate transaminase (AspB) or alanine transferase (AlaT, Marienhagen and Eggeling, 2008) thereby forming Asp or Ala, respectively. Accordingly, Asp and Ala uptake rates are likely to be reduced as observed in the experiments. Ala itself may serve as amino donor for Val production via AvtA-reaction (Marienhagen and Eggeling, 2008) which finally links low Val, Ala, Asp, and Glu uptakes with tremendously high Gln consumption. For completion, Ala uptake via MetP (Trötschel et al., 2008; **Supplementary Table S1**) may have been hampered further by competition with Met, while Lange et al. (2003) showed that *C. glutamicum* WT cannot use Val as sole carbon or nitrogen source if supplemented in CGXII shaking flask experiments. Similar to Gln, the immense Asn uptake likewise reduced the uptake of other AAs of the Asp-family. Aparaginase AnsA which is suspected to act as an overflow enzyme (Mesas et al., 1990) might have been active to hydrolyze Asn to Asp and ammonia thereby minimizing the need for Asp uptake. Besides, Ziert (2014) showed that Asp is a poor nitrogen source, is even less attractive as carbon source, and was therefore not consumed in batch Asp supplementation experiments conducted in microflower plates. Interesting enough, Asp is a precursor for Lys biosynthesis which may explain why immense Asn uptake and its conversion to Asp may finally reduce Lys uptake to 26% of the biomass needs. The corresponding consumption rate (0.025 mmol g^-1^ h^-1^) was in the same range as the Lys uptake determined in isotopic studies by Seep-Feldhaus et al. (1991) (0.015 mmol g^-1^ h^-1^). Both rates are significantly lower than the rate needed to satisfy the composition demand at the measured growth rate of 0.468 h^-1^ (0.099 mmol g^-1^ h^-1^). Summarizing, the immense uptake of Gln and Asn was likely to minimize the uptakes of Ala, Asp, Glu, Lys, and Val, which is also reflected by FBA assuming unlimited uptake of one single AA. In particular, Asn, Asp, Gln, and Glu, the protagonists of the transaminase network, revealed maximum (theoretical) uptake rates in addition to glucose (**Figure 4C**).

So far, no clear experimental observations give evidence on crucial Pro consumption (Bott and Niebisch, 2003; Jensen and Wendisch, 2013) although *putA* (*cg0129*) has been annotated in the genome of *C. glutamicum* encoding proline dehydrogenase which would ultimately provide Glu and NADH (Ling et al., 1994). Besides, the low consumption of Gly (14% with respect to the biomass composition) might be explained by its intracellular production via serine hydroxymethyltransferase being a by-product of 5,10-methylenetetrahydrofolate (MTHF) synthesis (Simic et al., 2002). FBA simulations supported the hypothesis: unlimited Gly uptake as a co-substrate of limited glucose uptake revealed that only 60% of the consumed Gly replenished the *in silico* cell’s demand for Gly, while the remaining amount was available as by-product of the MTHF pathway.

## Conclusion

In this two-staged study, physiological investigations were conducted to improve growth and nutrient consumption of the industrial production host *C. glutamicum*. Supplementation of minimal medium with complex compounds (BHI) relieved the strain of energy intensive biosynthesis, most likely of proteins and fatty acids, subsequently reducing the ATP demand of the cell. In turn, growth was accelerated to the WT strain’s apparent maximum of 0.67 h^-1^ in 37 g BHI L^-1^, while the TC consumption was proportionally increased. However, glucose consumption was only stimulated slightly under certain circumstances (1–10 g BHI L^-1^) and revealed falling tendencies in rich environments. Besides, biomass carbon yields deduced from BHI and AA supplementation experiments revealed nutrient-irrespective limits of about 18 g C-mol^-1^ that may be a target of further strain engineering studies. Experimental consumption rates of 18 AAs under process-relevant conditions were reported, and Asn and Gln were identified as main protagonists due to their role in the transaminase network of *C. glutamicum*. FBA simulations with *iMG481* confirmed the experimental findings. Summarizing, the revealed AA uptake kinetics, the ranking of individual AA needs, observed AA exchange abilities, the properties of BHI supplementation, and the unraveled apparent limit of carbon-to-biomass conversion offer a highly fruitful basis for improving process productivities and for metabolic engineering. Such studies may expand the application of *C. glutamicum* not only as an AAs producer but also for the production of rather growth coupled products such as technical proteins.

## Author Contributions

MG designed the study, carried out the bioreactor experiments, analyzed the datasets, and drafted the manuscript. MG and JZ expanded the stoichiometric network model and performed the flux balance analyses. TH and AN analyzed the datasets and corrected the manuscript. BB and RT conceived the whole study and corrected the manuscript. All authors read and approved the final manuscript.

## Conflict of Interest Statement

The authors declare that the research was conducted in the absence of any commercial or financial relationships that could be construed as a potential conflict of interest.
