# Supplementary material for: Physiological Response of Corynebacterium glutamicum to Increasingly Nutrient-Rich Growth Conditions
Source: Front Microbiol. 2018 Aug 29;9:2058. doi: 10.3389/fmicb.2018.02058 (PMC6123352; doi:10.3389/fmicb.2018.02058)
Supplement: Supplementary file 3 [file Data_Sheet_1.DOCX]

Supplementary Material: supplementary figure 1 and table 1

Physiological response of *Corynebacterium glutamicum* to increasingly nutrient-rich growth conditions

Michaela Graf, Julia Zieringer, Thorsten Haas, Alexander Nieß, Bastian Blombach, Ralf Takors^*^

Institute of Biochemical Engineering, University of Stuttgart, Stuttgart, Germany

*** Correspondence:**Prof. Dr.-Ing. Ralf Takors
takors@ibvt.uni-stuttgart.de

#
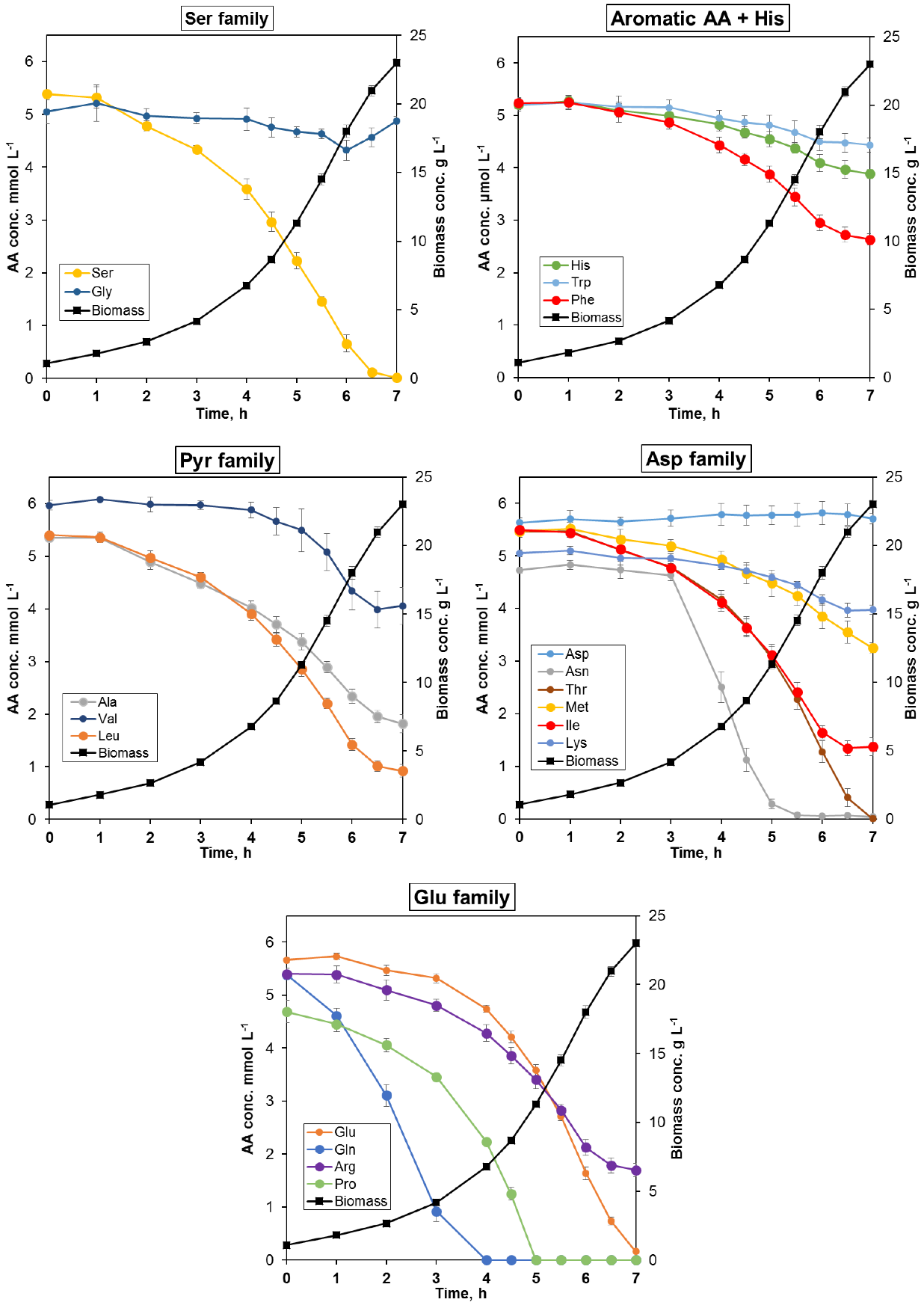
Figures

**Supplementary figure 1.** Amino acid (AA) consumption during bioreactor batch processes with
*C. glutamicum* WT cultivated in CGXII minimal medium supplemented with 4 % (w/v) glucose and 5 mM of all essential AA except L-cysteine and L-tyrosine. Values represent the statistical mean ± standard deviation of three individual bioprocesses.

# Tables

**Supplementary table 1.** Summary of amino acid (AA) importers of *C. glutamicum* used as basis for AA uptake studies with stoichiometric network model *iMG481*. The experimental consumption rate (q_AA_), and maximum uptake rates (v_max_) and the saturation constants (K_M_) experimentally determined for the respective transporters are listed with corresponding references. AA transporters active under process conditions (cf. supplementary figure 1) are marked with *. n.c.d.: no consumption detected; n.v.: no value available

| **AA** | **Experiment** | **Literature values** | | **Transporter name, type, (and additional information)** | **Reference** |
| --- | --- | --- | --- | --- | --- |
|  | **q_AA_, mmol g^-1^ h^-1^** | **v_max_, mmol g^-1^ h^-1^** | **Km, µmol L^-1^** |  |  |
| **Ala** | 0.083 ± 0.002 | 0.198 ± 0.012 | 69 ± 11 | MetP*, secondary | Trötschel et al. 2008 |
| **Arg** | 0.093 ± 0.003 | n. d. a. |  |  |  |
| **Asn** | 0.302 ± 0.003 | n. d. a. |  |  |  |
| **Asp** | n. c. d. | 0.456 | 1 | Uptake by primary Glu ATP*-dependent transporter | Krämer et al. 1990, |
| **Gln** | 0.694 ± 0.005 | 0.750 | 36 | Secondary* | Siewe et al. 1995 |
| **Glu** | 0.117 ± 0.001 | 0.900 | 600 | GluS*, secondary | Burkovski et al. 1996 |
|  |  | 0.090 | 0.45 (Glc grown); 0.9 (Ace grown); 1.35 (Glu grown) | Primary | Krämer et al. 1990 |
| **Gly** | 0.025 ± 0.006 | n. d. a. |  |  |  |
| **His** | 0.031 ± 0.002 | 0.2022 ± 0.026 | 11.4 ± 2.03 | AroP*, secondary | Shang et al. 2013 |
|  |  | 0.016 ± 0.004 |  | Uptake in presence of Trp, Tyr, Phe, His |  |
| **Ile** | 0.108 ± 0.002 | 0.066 | 5.4 | BrnQ*, secondary | Ebbighausen et al. 1989 |
|  |  | 0.0624 ± 0.007 |  | BrnQ*, secondary | Tauch et al. 1998 |
| **Leu** | 0.112 ± 0.002 | 0.056 | 9 | BrnQ*, secondary | Ebbighausen et al. 1989 |
|  |  |  |  |  |  |
| **AA** | **Experiment** | **Literature value** | | **Transporter name, type, (and additional information)** | **Reference** |
|  | **q_AA_, mmol g^-1^ h^-1^** | **v_max_, mmol g^-1^ h^-1^** | **Km, µmol L^-1^** |  |  |
| **Lys** | 0.025 ± 0.001 | 0.009 | 10 | LysI*, Lys-Lys/Ala/Ile/Val antiporter | Bröer & Krämer 1990,  Seep-Feldhaus et al. 1991 |
| **Met** | 0.046 ± 0.002 | 0.0318 ± 0.005 | 0.1 | MetNIQ, ABC-type | Trötschel et al. 2008 |
|  |  | 0.042 ± 0.003 | 0.2 ± 0.09 | Data for *C. glutamicum* ΔmetP |  |
|  |  | 0.109 ± 0.008 | 53 ± 16 | MetP*, secondary |  |
|  |  | 0.085 ± 0.003 | 65.6 ± 6.7 | Data for *C. glutamicum* ΔmetNIQ |  |
| **Phe** | 0.066 ± 0.002 | 0.024 ± 0.001 |  | AroP*, secondary | Wehrmann et al. 1995 |
|  |  | 0.0287 ± 0.005 |  | AroP, in presence of Trp, Tyr, Phe, His | Shang et al. 2013 |
|  |  | 0.072 ± 0.006 | 10.4 ± 1.5 | PheP | Zhao et al. 2011 |
| **Pro** | 0.218 ± 0.010 | 1.200 | 7.6 | PutP*, expressed in *Escherichia coli* | Peter et al. 1997 |
|  |  | 0.720 | n. d. a. | ProP*, EctP* (basal) | Weinand et al. 2007 |
| **Ser** | 0.134 ± 0.003 | n. d. a. |  |  |  |
| **Thr** | 0.118 ± 0.001 | 0.270 ± 0.030 | 6.78 ± 0.7 | Secondary*, *C. glutamicum* MH20-22B (Lys producer) | Palmieri et al. 1996 |
| **Trp** | 0.022 ± 0.001 | 0.013 ± 0.007 | n. d. a. | AroP*, secondary | Wehrmann et al. 1995 |
|  |  | 0.052 ± 0.002 | n. d. a. | AroP, secondary, in the presence of Trp, Tyr, Phe, His | Shang et al. 2013 |
| **Val** | 0.051 ± 0.008 | 0.078 | 9.5 | BrnQ*, secondary | Ebbighausen et al. 1989 |

# References for supplementary table 1

Bröer, S., and Krämer, R. (1990). Lysine uptake and exchange in *Corynebacterium glutamicum*. *Journal of bacteriology*, 172(12), 7241-7248. doi: 10.1128/jb.172.12.7241-7248.1990

Burkovski, A., Weil, B., and Krämer, R. (1996). Characterization of a secondary uptake system for L‐glutamate in *Corynebacterium glutamicum*. *FEMS microbiology letters*, 136(2), 169-173. doi: 10.1111/j.1574-6968.1996.tb08044.x

Ebbighausen, H., Weil, B., and Krämer, R. (1989). Transport of branched-chain amino acids in *Corynebacterium glutamicum*. *Archives of microbiology*, 151(3), 238-244. doi: 10.1007/BF00413136

Krämer R., Lambert, C., Hoischen, C. and Ebbighausen, H. (1990), Uptake of glutamate in *Corynebacterium glutamicum*. *European Journal of Biochemistry*, 194: 929–935. doi: 10.1111/j.1432-1033.1990.tb19488.x

Marx, A., de Graaf, A. A., Wiechert, W., Eggeling, L., and Sahm, H. (1996). Determination of the fluxes in the central metabolism of *Corynebacterium glutamicum* by nuclear magnetic resonance spectroscopy combined with metabolite balancing. *Biotechnology and Bioengineering*, 49(2), 111-129. doi: 10.1002/(SICI)1097-0290(19960120)49:2<111::AID-BIT1>3.0.CO;2-T

Palmieri, L., Berns, D., Krämer, R., and Eikmanns, M. (1996). Threonine diffusion and threonine transport in *Corynebacterium glutamicum* and their role in threonine production. *Archives of microbiology*, 165(1), 48-54. doi: 10.1007/s002030050295

Peter, H., Bader, A., Burkovski, A., Lambert, C., and Krämer, R. (1997). Isolation of the *putP* gene of *Corynebacterium glutamicum* and characterization of a low-affinity uptake system for compatible solutes. *Archives of microbiology*, 168(2), 143-151. doi: 10.1007/s002030050480

Siewe, R. M., Weil, B., and Krämer, R. (1995). Glutamine uptake by a sodium-dependent secondary transport system in *Corynebacterium glutamicum*. *Archives of microbiology*, 164(2), 98-103. doi: 10.1007/BF02525314

Shang, X., Zhang, Y., Zhang, G., Chai, X., Deng, A., Liang, Y., and Wen, T. (2013). Characterization and molecular mechanism of AroP as an aromatic amino acid and histidine transporter in *Corynebacterium glutamicum*. *Journal of bacteriology*, 195(23), 5334-5342. doi: 10.1128/JB.00971-13

Tauch, A., Hermann, T., Burkovski, A., Krämer, R., Pühler, A., and Kalinowski, J. (1998). Isoleucine uptake in *Corynebacterium glutamicum* ATCC 13032 is directed by the *brnQ* gene product. *Archives of microbiology*, 169(4), 303-312. doi: 10.1007/s002030050576

Trötschel, C., Follmann, M., Nettekoven, J. A., Mohrbach, T., Forrest, L. R., Burkovski, A. et al. (2008). Methionine uptake in *Corynebacterium glutamicum* by MetQNI and by MetPS, a novel methionine and alanine importer of the NSS neurotransmitter transporter family. *Biochemistry*, 47(48), 12698-12709. doi: 10.1021/bi801206t

Wehrmann, A., Morakkabati, S., Krämer, R., Sahm, H., and Eggeling, L. (1995). Functional analysis of sequences adjacent to *dapE* of *Corynebacterium glutamicum* reveals the presence of *aroP*, which encodes the aromatic amino acid transporter. *Journal of bacteriology*, 177(20), 5991-5993. doi: 10.1128/jb.177.20.5991-5993.1995

Weinand, M., Krämer, R., and Morbach, S. (2007). Characterization of compatible solute transporter multiplicity in *Corynebacterium glutamicum*. *Applied microbiology and biotechnology*, 76(3), 701-708. doi: 10.1007/s00253-007-0938-4

Zhao, Z., Ding, J. Y., Li, T., Zhou, N. Y., and Liu, S. J. (2011). The ncgl1108 (PheP Cg) gene encodes a new L-Phe transporter in *Corynebacterium glutamicum*. *Applied microbiology and biotechnology*, 90(6), 2005. doi: 10.1007/s00253-011-3245-z
